# Balancing Inflammation: The Link between Th17 and Regulatory T Cells

**DOI:** 10.1155/2016/6309219

**Published:** 2016-06-19

**Authors:** Maggie L. Diller, Ragini R. Kudchadkar, Keith A. Delman, David H. Lawson, Mandy L. Ford

**Affiliations:** ^1^Department of Surgery of Emory University, 1364 Clifton Road, Atlanta, GA 30322, USA; ^2^Department of Hematology and Medical Oncology, Winship Cancer Institute of Emory University, 1365 Clifton Road No. C, Atlanta, GA 30322, USA; ^3^Department of Surgical Oncology, Winship Cancer Institute of Emory University, 1365 Clifton Road No. C, Atlanta, GA 30322, USA; ^4^Emory Transplant Center of Emory University, 5105 Woodruff Memorial Research Building, 101 Woodruff Circle, Atlanta, GA 30322, USA

## Abstract

CD4^+^ T cell compartments in mouse and man are composed of multiple distinct subsets each possessing unique phenotypic and functional characteristics. IL-17-producing CD4^+^ T cells (Th17 cells) represent a distinct subset of the CD4^+^ T cell lineage. Recent evidence suggests that Th17 cells carry out effector functions similar to cytotoxic CD8^+^ T cells and play an important role in the clearance of extracellular pathogens and fungi. Th17 cell differentiation and function are closely related to the development and function of regulatory T cells (T_REG_). The balance between these two cell populations is essential for immune homeostasis and dysregulation of this balance has been implicated in a variety of inflammatory conditions including autoimmunity, allograft rejection, and tumorigenesis. Emerging evidence reports a significant amount of plasticity between the Th17 and regulatory T cell compartments, and the mechanisms by which these cells communicate and influence each other are just beginning to be understood. In this review, we highlight recent findings detailing the mechanisms driving Th17 and T_REG_ plasticity and discuss the biologic consequences of their unique relationship.

## 1. Introduction

CD4^+^ T cells represent an important arm of the adaptive immune response and upon activation differentiate into a variety of subsets including Th1 and Th2 cells, follicular helper (Tfh) cells, Th17 cells, and regulatory T cells (T_REG_). The functions of the CD4^+^ T cell compartment are diverse, ranging between activation of both immune and nonimmune cells, direct cytolytic activity, and dampening of the immune response [1]. While naïve CD4^+^ T cell differentiation was previously thought to involve commitment to a specific subset lineage, more recent data has identified significant plasticity within the CD4^+^ compartment [2]. In particular, recent studies have identified significant flexibility between the Th17 and T_REG_ compartments. Th17 cells are a distinct CD4^+^ effector lineage and play important roles in host defense against a variety of pathogens as well as in the pathogenesis of several inflammatory conditions. While regulatory T cells have been shown to attenuate both Th1 and Th2 responses, their impact on Th17 cell function is less clear. In fact, the differentiation of Th17 cells appears to be closely linked to the differentiation of T_REG_ [3]. Both cell populations require TGF-*β* for differentiation [3], and in vivo studies have identified a subset of CD4^+^ T cells that dually express elements of both the T_REG_ and Th17 phenotypes (Diller et al. manuscript submitted) [3, 4]. This paper will focus on the mechanisms driving differentiation and development of Th17 and regulatory T cells and the functional implications of their uniquely flexible relationship.

## 2. TGF-***β*** Is Critical for Both Th17 and T_**REG**_ Development

**Figure 1:**
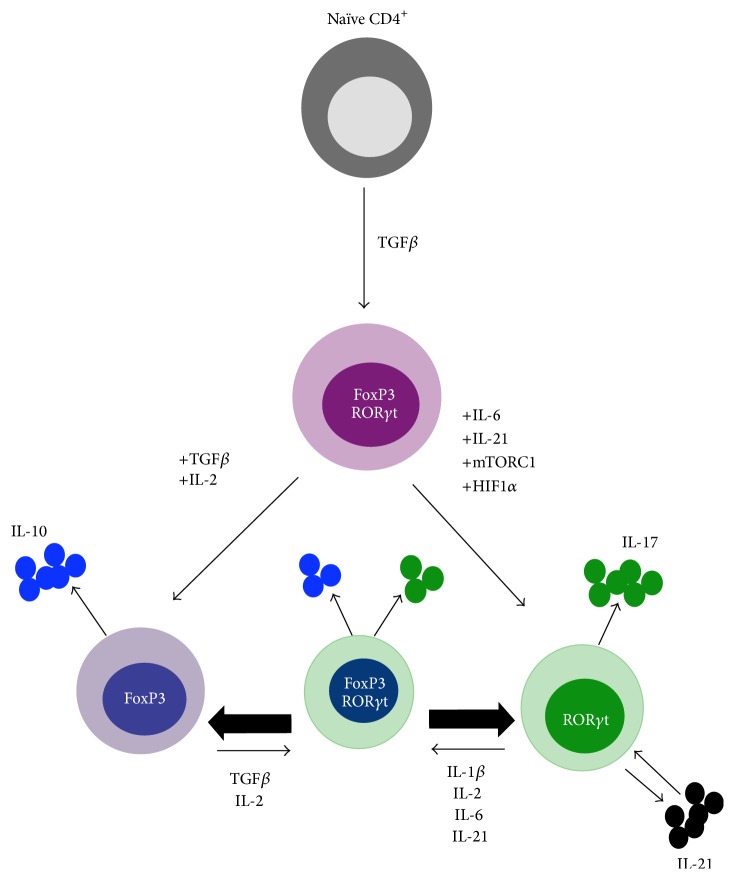
Upon interaction with TGF-*β* within the periphery, naïve CD4^+^ T cells upregulate the transcription factors for both Th17 cells (ROR*γ*t) and regulatory T cells (FoxP3). Differentiation of either lineage depends on a multitude of factors including the surrounding cytokine environment, metabolic signalling pathways, and epigenetic modifications. These internal and external cues function together to allow for a uniquely plastic relationship whereby transdifferentiation of Th17 cells and T_REG_ can occur.

All naïve CD4^+^ T cells share an initial pathway of activation, signalling, through the T cell receptor (TCR) and costimulatory molecules induced the production of IL-2 leading to activation of STAT5 and entry into the cell cycle. From here, lineage specific factors drive the differentiation of distinct cell subsets. Both Th17 cells and peripherally induced T_REG_ require TGF-*β* for differentiation and development, introducing an elegant mechanism by which these two compartments interrelate (Figure 1). While natural T_REG_  (nT_REG_) develop from the thymus and are TGF*β*-independent, induced T_REG_  (iT_REG_) stem from extrathymic, naïve T cell precursors and are TGF-*β*-dependent [5]. TGF-*β* promotes Th17 and iT_REG_ development by inducing the expression of the transcription factors retinoic-acid-receptor-related orphan receptor *γ*t (ROR*γ*t) and fork-head box P3 (FoxP3), respectively. Whether cells are shuttled towards a proinflammatory Th17 phenotype or a regulatory phenotype depends largely on the surrounding cytokine environment (Figure 1).

IL-6 has been identified as an important mediator driving the development of Th17 cells via activation of STAT3 [6–8]. In vitro and in vivo studies demonstrated that upon initial interaction with TGF-*β* naïve CD4^+^ T cells upregulate both ROR*γ*t and FoxP3 [3, 9, 10]. Zhou et al. showed that in this setting of cotransduction FoxP3 initially inhibits Th17 differentiation by physically binding to ROR*γ*t [3]. In the absence of IL-6 and other proinflammatory cytokines, TGF-*β* reinforces FoxP3-mediated inhibition of ROR*γ*t and favors development of the T_REG_ lineage [3]. In the presence of IL-6, STAT3 activation releases FoxP3 inhibition and the receptor for IL-23 (IL-23R) is upregulated, inducing Th17 differentiation [3]. While APC-derived IL-23 plays a less crucial role in the initiation of Th17 differentiation, it is critical for the expansion and maintenance of the Th17-committed lineage, further activating STAT3 and dampening IL-10 production [8, 11]. It is important to note that TGF-*β*-induced Th17 differentiation can occur in the absence of IL-6 provided there is sufficient IL-21 present. Human T cells treated ex vivo with IL-21 and TGF-*β* led to an upregulation of IL-23R and inhibition of FoxP3 expression via induction of ROR*γ*t, an effect similar to that seen with IL-6 and TGF-*β* [12]. Upon differentiation, Th17 began secreting IL-21 which functions in an autocrine loop to further promote Th17 development [13, 14].

## 3. Mediators of Metabolism Help Shape the Balance between Th17 and Regulatory T Cells

In addition to the surrounding cytokine milieu, T cell metabolic reprogramming and the external cues signalling metabolic pathways serve as important regulators of the balance between Th17 cells and T_REG_. Naïve T cells have a relatively low energy demand and therefore utilize glucose oxidation via the tricarboxylic acid (TCA) cycle and the oxidation of fatty acids to meet energy requirements [15]. Memory T cells and T_REG_ have similar energy demands and metabolic profiles to those of naïve T cells and are metabolically distinct from effector T cells [15]. Upon T cell activation, cells augment their metabolic program in order to meet the demands of cell proliferation and growth and to fuel the synthesis of cytokines, macromolecules, and intracellular intermediates [16]. This metabolic reprogramming involves downregulation of lipid oxidation and an increase in glucose utilization via aerobic glycolysis and glutamine catabolism [17].

The impact of metabolic reprogramming on T cell fate and function was largely discovered through the investigation of mTOR. mTOR serves as a central environmental sensor, integrating signals such as growth factors, nutrients, oxygen, and energy levels in order to orchestrate multiple cell functions [18, 19]. Under steady state conditions, mTOR is inhibited; however, upon antigen recognition by naïve T cells, mTOR is activated, serving as a central regulator driving T cell differentiation and function [15, 20]. mTOR exists as two multiprotein complexes: mTOR complex 1 (mTORC1) and mTOR complex 2 (mTORC2); and optimal activation of these complexes results in the upregulation of glycolysis and activation of stat signalling needed to support differentiation into specific effector lineages.

Naïve CD4^+^ T cells that lack both mTORC1 and mTORC2 fail to upregulate the necessary glycolytic machinery to support effector function and instead take on a regulatory phenotype [15]. Pharmacologic inhibition of mTOR further exemplifies the opposing roles of mTOR in effector versus regulatory T cell fate and function. Administration of rapamycin, an mTOR inhibitor, induces de novo FoxP3 expression and also expands preexisting nT_REG_ [21, 22]. Lack of mTOR activation impacts the balance between regulatory and Th17 cells specifically by increasing T cell sensitivity to TGF-*β*, overcoming the activating effects of proinflammatory cytokines on STAT3 [23]. Follow-up studies utilizing a human model of transplantation demonstrated that administration of rapamycin exerted its regulatory effects by inhibiting STAT3 and thus preventing development of the Th17 lineage specifically while promoting T_REG_ development [24]. The effects of mTOR inhibition on the T_REG_ : Th17 balance held true under Th17 polarizing conditions in which human PBMCs were cultured ex vivo with IL-6, IL-23, and IL-1*β* [24].

While complete inhibition of mTOR shifts naïve CD4^+^ T cells away from a Th17 phenotype and towards a regulatory phenotype, blockade of the mTOR complexes individually yields different results. mTORC1 appears to be principally important in the generation of Th17 cells. Mice lacking mTORC1 activity fail to generate Th17 responses [25, 26]. Conversely, when mTORC1 activity is maintained and mTORC2 activity is suppressed, Th17 responses are preserved [15, 19, 26]. The ability of mTOR and mTORC1 specifically to generate the Th17 cell lineage stems in part from its ability to induce hypoxia inducible factor 1*α* (HIF1*α*). HIF1*α* is a critical sensor of hypoxia and is responsible for initiating the cell response to low oxygen levels. Importantly, many nonhypoxic stimuli serve to upregulate HIF1*α*, including TCR activation [27]. HIF1*α* activates genes involved in glycolysis and promotes upregulation of glucose metabolism. As such, HIF1*α* serves as a critical mediator of Th17 development.

Th17 cells have been shown to rely more heavily on glycolytic pathways than any other T cell subset [15]. Because of its importance in the upregulation of glycolytic machinery, HIF1*α* is highly expressed in cells committed to the Th17 lineage [28, 29]. HIF1*α* directly promotes Th17 differentiation via activation of ROR*γ*t and regulation of Th17 signature genes [29]. It was recently discovered that HIF1*α* also functions to increase the microRNA miR-210, a signature of hypoxia, and this molecule is highly expressed in Th17 cells [30]. Hypoxia synergizes with TCR and CD28 signalling to increase expression of miR-210 which subsequently functions to inhibit HIF1*α* in a negative feedback loop [30]. In concert with Th17 differentiation, HIF1*α* attenuates T_REG_ development by mediating FoxP3 degradation via proteasomal degradation pathways, occurring under both normoxic and hypoxic conditions [29]. As a result, HIF1*α* represents another key player in generating an inflammatory environment via its direct effects on both Th17 and regulatory T cells.

Endogenous and environmental metabolites and toxins also mediate differentiation along the Th17 and T_REG_ axis, in particular via their effects on the nuclear receptor aryl hydrocarbon receptor (AHR). AHR is critical to protecting hosts from environmental toxins and is activated by external toxins such as tetrachlorodibenzo-p-dioxin (TCDD) and endogenous ligands such as 6-formylindolo[3,2-b]carbazole (FICZ) (a metabolite of tryptophan) [31]. Recent studies have implicated AHR activation in both Th17 and T_REG_ development, depending on the activating ligand [31]. Studies treating both human and mouse CD4^+^ T cells in vitro with FICZ enhanced IL-17 and IL-22 expression via activation of AHR [32]. AHR appears to support Th17 differentiation via its direct interaction with the Th17-inhibitory STAT1 [33]. Mechanisms underlying AHR induced T_REG_ expansion are less clear; however, studies have shown that external toxins such as TCDD can generate human T_REG_ in vitro and serve as a substitute for TGF-*β* under certain conditions [34]. The ligand-specific effect of AHR activation on Th17/T_REG_ development offers a unique target for therapeutic intervention, and the mechanisms behind this receptor's differential effects are an important area of ongoing study.

## 4. Epigenetic Processes Control Th17 and T_**REG**_ Differentiation and Allow for Subset Redirection 

Launching the differentiation of a specific T cell lineage requires the conversion of cell extrinsic information into cell intrinsic changes resulting in the augmentation of gene expression patterns. Epigenetic processes allow for precise control of gene expression, including imprinted control of induced genetic programs in response to changing environmental cues. Epigenetic processes do not induce changes in the sequence of the DNA but instead involve modifications such as DNA methylation and histone acetylation which helps determine the gene expression patterns of a given cell. The changes induced are phenotypic rather than genotypic; thus epigenetic modifications and the information they encode can be heritable but remain malleable [35].

Genomewide chromatin immunoprecipitation studies (CHIP) have identified specific histone modifications associated with the activation and repression of genes within CD4^+^ T cells. The presence of both types of histone modifications, termed bivalency, allows for a gene promoter to become activated or silenced and is necessary for subset plasticity. The Th17-specific transcription factor, ROR*γ*t, carries bivalent epigenetic modifications, supporting the observed capacity for subset redirection [36]. The Th17 lineage also demonstrates marked DNA demethylation in the promoter regions of* Il17a*,* Il17f*,* and RAR-related orphan receptor C *(*RORC*) [37, 38]. A genomewide analysis of changes in the DNA methylation patterns of naïve CD4^+^ T cells during subset differentiation revealed that Th17 cells are more similar to naïve CD4^+^ T cells than Th1 cells [39]. Furthermore, Th17 cells were found to display an even higher number of demethylated regions when compared to naïve CD4^+^ T cells, suggesting that these processes contribute to the marked plasticity observed in the Th17 compartment [39].

In contrast to Th17 cells which represent a relatively unstable T cell population, regulatory T cells are generally stable under normal conditions [40]. Miyao et al. concluded that T_REG_ exist in a “committed state” secondary to specific epigenetic modifications of the FoxP3 locus [41]. Genetic fate mapping, which permanently marks FoxP3^+^ cells and their progeny, has shown that FoxP3^+^CD4^+^ T cells are capable of transiently losing their FoxP3 expression (termed “exFoxP3 cells”) [42, 43]. Miyao et al. identified a subset of exFoxP3 cells, “latent” T_REG_, which retained their regulatory memory after downregulation of FoxP3 and robustly reexpressed FoxP3 and suppressive function upon activation [41]. Conversely, a subpopulation of exFoxP3 cells was characterized by a fully methylated TSDR and was unable to reexpress FoxP3 or reacquire regulatory function [41]. While there remains some controversy regarding the phenotypic and functional plasticity of exFoxP3 cells (discussed below), the epigenetic processes guiding the differentiation of Th17 and regulatory T cells play an important role in regulating the relationship between these two compartments.

## 5. Th17 and Regulatory T Cells Represent Highly Plastic Compartments and Are Capable of Transdifferentiation

IL-2, a potent growth factor for both effector and regulatory T cells, has previously been shown to potentiate the indirect relationship that exists between regulatory and Th17 cells [44, 45]. Studies utilizing mouse models have demonstrated that IL-2 inhibits Th17 expansion via a STAT5 mechanism [44]. Additionally, regulatory T cells induced by TGF-*β* in the presence of IL-2 are resistant to Th17 conversion by IL-6 during in vitro cell cultures [46]. However, more recent data calls into question the dichotomous effect of IL-2 on the Th17 and T_REG_ compartments, and there is mounting evidence to suggest that IL-2 may promote the conversion of T_REG_ into Th17 cells. In a human model of uveitis and scleritis, IL-17 expression increased after in vitro stimulation with IL-2, explaining in part the effectiveness of IL-2R blockade in the treatment of certain autoimmune diseases [47]. In an in vivo model of human melanoma, administration of high dose IL-2 (HDIL-2) led to expansion of both the Th17 and regulatory T cell compartments, demonstrating increased cell counts and frequencies early in the course of treatment [48].

Transdifferentiation of regulatory T cells into Th17 cells may in part explain the unexpected stimulatory effect of IL-2 on the Th17 compartment. In vitro assays have demonstrated that T_REG_ stimulated under Th17 polarizing conditions in the presence of exogenous IL-2 can be converted into IL-17 expressing CD4^+^ T cells [49]. The proposed mechanism for T_REG_ and Th17 interconversion in this model was dependent on IL-1*β*, a cytokine produced along with IL-6 by activated monocytes. IL-1*β* was shown to induce downregulation of FoxP3 as well as inhibit T_REG_ suppressive function [49]. Support for this hypothesis can be drawn from in vivo human models. FoxP3^+^IL-17^+^CD4^+^ T cells were present in the peripheral blood of melanoma patients undergoing systemic IL-2 therapy (Diller et al. manuscript submitted). More importantly, this cell population coincided with peak T_REG_ frequencies and immediately preceded peak Th17 frequencies (Diller et al. manuscript submitted).

As mentioned above, genetic fate mapping has led to the identification of exFoxP3 cells and offers further support for the proposed plasticity of FoxP3^+^CD4^+^ T cells. Zhou et al. demonstrated that exFoxP3 T cells developed from both natural and induced T_REG_ and exhibited an activated memory cell phenotype, secreting the inflammatory cytokines IFN-*γ* and IL-17 [42]. Using similar techniques, Komatsu et al. identified IL-17-expressing exFoxP3 (exFoxP3 Th17) cells as important mediators of inflammation in a mouse model of autoimmune arthritis, demonstrating enhanced osteoclastogenic ability when compared to traditionally derived Th17 cells [50].

While these studies have shown that FoxP3^+^CD4^+^ T cells are capable of transiently losing their FoxP3 expression and go on to acquire inflammatory function, the findings presented by Miyao et al. indicate that exFoxP3 T cells consist of two distinct categories: those that acquire an inflammatory phenotype versus those that retain their FoxP3 memory (“latent” T_REG_). Miyao et al. concluded that T_REG_ represent a stable cell lineage, distinct from the subpopulation of exFoxP3 cells which irreversibly lose their T_REG_ function and acquire a pathogenic phenotype [41]. This point remains controversial and has been difficult to investigate fully due to significant instability during in vitro restimulation of Th17 cells. Utilizing a new model of fate mapping which enabled analysis of cells expressing IL-17A, IL-10, and FoxP3 without restimulation, Gagliani et al. circumvented this issue and found that CD4^+^ T cells previously expressing IL-17A go on to acquire an anti-inflammatory phenotype [51]. Acquisition of a regulatory phenotype was determined by changes in their signature transcriptional profile and the acquisition of potent suppressive functions, including the ability to prevent Th17-mediated colitis in a mouse model [51].

## 6. The Th17 : T_**REG**_ Balance Plays a Central Role in Disease Pathogenesis

Th17 and regulatory T cells represent two arms of an immune response, and their uniquely plastic relationship dictates the flavor of their surrounding immune environment, allowing for shifts between pro- and anti-inflammatory states. As such, the balance between these two compartments is central to the pathogenesis of various diseases and conditions including but not limited to autoimmunity, transplant rejection, and carcinogenesis.

The pathogenic role for Th17 cells was first highlighted by studies involving animal models of experimental autoimmune encephalitis (EAE) [52]. Mice deficient in the receptor for the Th1 effector cytokine IFN*γ* developed enhanced EAE [53]. Further experiments identified IL-23-driven Th17 cells as central mediators of tissue damage in autoimmunity [54]. Both IL-23 and IL-17 defective mice show reduced susceptibility to autoimmune and chronic inflammatory diseases [55]. IL-17 has since been shown to be elevated in patients with rheumatoid arthritis, multiples sclerosis, inflammatory bowel disease, and psoriasis [52, 56, 57]. Diminished T_REG_ counts and suppressor function often accompany Th17-mediated autoimmunity, propagating inflammation and tissue destruction. Th17 : T_REG_ ratios are elevated in patients with active rheumatoid arthritis compared to healthy controls, highlighting a role for Th17 : T_REG_ imbalance in autoimmune-related pathology [58].

Th17-mediated inflammation appears to play an important role in both acute and chronic allograft rejection. IL-17 antagonism in a rat cardiac allograft model prolonged graft survival, and in experimental models of lung transplantation rejection is associated with increased IL-17 and IL-23 transcripts at the site of rejection and within draining lymph nodes [59–61]. Furthermore, in addition to propagating an inflammatory cytokine milieu, Th17 cells are also responsible for neutrophilic recruitment and allograft infiltration, an additional mechanism contributing to transplant rejection [60].

Host metabolic conditions as well as substrate availability may serve as important factors generating the Th17 phenotype and driving Th17-mediated inflammation in transplant rejection. Yuan et al. found that hyperlipidemic mice demonstrated accelerated allograft rejection and this was associated with increased serum levels of IL-2, IL-6, and IL-17 [62]. Hyperlipidemic mice demonstrated increased numbers of Th17 cells in the periphery and in rejecting allographs when compared to controls [62]. The inflammatory signals present in a rejecting allograft further propagate Th17-mediated inflammation through the induction of T_REG_-Th17 interconversion, and the plasticity between the T_REG_ and Th17 compartments poses a significant problem for T_REG_ mediated transplantation tolerance. Benghiat et al. showed that T cell mediated rejection became Th17 biased upon adoptive cotransfer of T_REG_ with naïve monospecific antidonor T cells [60]. Therefore, targeting in vivo inflammatory signals in concert with the administration or induction of T_REG_ will likely be required to achieve desired results [63]. In all, a multitude of host factors contributing to Th17-mediated rejection offer new points of intervention for decreasing inflammation and enhancing graft survival.

While the role of Th17 cells in inflammation and autoimmunity is relatively well established, their function in tumor immunity continues to be strongly debated [64–66]. Studies examining the capacity of Th17 cells to promote or suppress tumor growth directly have been conflicting. Proinflammatory cytokines secreted by Th17 cells such as IL-17A have been shown to impair immune surveillance and promote tumor growth [67]. Conversely, Th17 cells have also been reported to eradicate established melanoma tumors in mice [68]. It is important to note however that Th17-mediated tumor regression was shown to be critically dependent on IFN*γ* and not IL-17 [67]. Therefore, a potential hypothesis for the opposing effects of Th17 cells on tumor growth is that different types of tumors may induce the differentiation of phenotypically distinct Th17 cells [66]. For example, natural versus induced Th17 cells are regulated differently by Akt and mTOR pathways [69]. Therefore, the impact of a specific tumor on downstream signalling pathways would be critical in determining Th17 phenotype and function.

While Th17 cells themselves have been shown to demonstrate both pro- and antitumorigenic properties, the balance of Th17 to regulatory T cells appears critically important in the process of tumor formation and progression. In a small cohort of patients with advanced stage melanoma, Th17 cell counts and frequencies increased in response to systemic cytokine therapy regardless of response to treatment (Diller et al. manuscript submitted) [48]. However, the ratio of Th17 to regulatory T cells was closely associated with response, with high Th17 : T_REG_ ratios directly correlating with tumor regression [48]. A thorough understanding of the tumor and nontumor factors shaping the balance between Th17 and T_REG_ generates a variety of potential therapeutic targets and could lead to the development of improved vaccine and T cell-based therapies.

## 7. Conclusion 

Th17 cells represent a unique population of effector CD4^+^ T cells. They play an important role in a wide variety of host defense mechanisms and are central mediators in diseases of inflammation. Their relationship with regulatory T cells emphasizes the remarkably plastic nature of these cell subsets and brings to light novel mechanisms of T cell differentiation and intercompartment interactions. Factors driving Th17 development and those shaping the balance between Th17 and regulatory T cells have significant biological implications for the design and implementation of novel therapeutic interventions.
